# The Association Between Somatic Health, Autism Spectrum Disorder, and Autistic Traits

**DOI:** 10.1007/s10519-019-09986-3

**Published:** 2019-12-06

**Authors:** Pei-Yin Pan, Kristiina Tammimies, Sven Bölte

**Affiliations:** 1 Center of Neurodevelopmental Disorders (KIND), Centre for Psychiatry Research, Gävlegatan 22, 11330 Stockholm, Sweden; 2 Department of Women’s and Children’s Health, Karolinska Institutet & Stockholm Health Care Services, Region Stockholm, Gävlegatan 22, 11330 Stockholm, Sweden; 3 Child and Adolescent Psychiatry, Stockholm Health Care Services, Region Stockholm, Stockholm, Sweden; 4 Curtin Autism Research Group, School of Occupational Therapy, Social Work and Speech Pathology, Curtin University, Perth, WA Australia

**Keywords:** Autism spectrum disorder, ASD, Neurodevelopmental disorders, Autistic traits, Twins, Comorbidity, Health, Neurology

## Abstract

This study used a twin cohort to investigate the association of autism spectrum disorder (ASD) and autistic traits with somatic health. A total of 344 twins (172 pairs; mean age 15.56 ± 5.62 years) enriched for ASD and other neurodevelopmental conditions were examined. Medical history and current physical problems were collected with a validated questionnaire to determine twin’s somatic health. The Social Responsiveness Scale (SRS-2) was used to measure the participant’s severity of autistic traits. Identified somatic health issues with significant within-twin pair differences were tested in relation to both ASD diagnosis and autistic traits in a co-twin control model. Twins with ASD exhibited more neurological and immunological health problems compared to those without ASD (p = 0.005 and p = 0.004, respectively). The intra-pair differences of neurological conditions and SRS-2 score were significantly correlated in monozygotic twins differing for autism traits (r = 0.40, p = 0.001), while the correlation was not found for immunological problems. In addition, a conditional model for analysis of within-twin pair effects revealed an association between neurological problems and clinical ASD diagnosis (Odds ratio per neurological problem 3.15, p = 0.02), as well as autistic traits (β = 10.44, p = 0.006), after adjusting for possible effects of co-existing attention deficit hyperactivity disorder and general intellectual abilities. Our findings suggest that neurological problems are associated with autism, and that non-shared environmental factors contribute to the overlap for both clinical ASD and autistic traits. Further population-based twin studies are warranted to validate our results and examine in detailed the shared genetic and environmental contributions of neurological problems and ASD.

**Electronic supplementary material:**

The online version of this article (10.1007/s10519-019-09986-3) contains supplementary material, which is available to authorized users.

## Introduction

Autism spectrum disorder (ASD) is a neurodevelopmental condition characterized by childhood onset heterogeneous alterations of social communication and interaction alongside repetitive, restricted behaviors and interests causing functional impairment (American Psychiatric Association 2013; Bölte et al. 2019a). Converging evidence shows that the etiology of ASD is multifactorial with numerous susceptible genes and environmental factors contributing to its phenotypic expressions (Bölte et al. 2019b; Hertz-Picciotto et al. 2018; Vorstman et al. 2017). The complexity of autism causation is compounded by the frequent co-occurrence of other neurodevelopmental, psychiatric, and somatic conditions (Hirvikoski et al. 2016; Simonoff et al. 2008).

Regarding physical health, individuals with ASD have been reported to be particularly at risk for immune dysregulation, gastrointestinal (GI) symptoms, and neurological complications (Alabaf et al. 2018; Kohane et al. 2012). Additional research investigates disease risks in ASD and the general population for other physical systems, while the results are rather inconsistent or the evidence is rather modest, such as infectious diseases (Rosen et al. 2007; Sabourin et al. 2019), disturbances in metabolism (Orozco et al. 2019; Rangel-Huerta et al. 2019), obesity (de Vinck-Baroody et al. 2015; Dreyer Gillette et al. 2015), and mitochondrial dysfunctions (Griffiths and Levy 2017; Hollis et al. 2017). The co-existence of somatic health problems in ASD is associated with increased autism symptom severity and lower quality of life (Aldinger et al. 2015; Kuhlthau et al. 2018). Higher prevalence of somatic complications in ASD suggests the possibility of an underlying genetic and/or environmental perturbation involving multiple systems that impact on the likelihood of the emergence of both ASD and somatic health issues. If this is true, autistic individuals with co-occurring somatic conditions could be considered to qualify as stratification subgroups, where targeted biological intervention might be meaningful and realistic (Doshi-Velez et al. 2014; Loth et al. 2016). Nevertheless, except for some specific genetic disorders and findings of gene pleiotropy (Cohen et al. 2005; Vorstman et al. 2017), the mechanisms underlying the comorbidity between somatic health issues, ASD and autistic-like behaviors remain unclear.

Several models have been postulated to explain shared pathways for individuals with combined autism and somatic condition presentations. For example, altered synaptic plasticity and an imbalance between excitation and inhibition in a critical time window of neurodevelopment has been proposed to account for ASD and epilepsy overlaps (Jacob 2016). For autistic individuals with immune dysregulation and abnormal cytokine profiles, neuropoietic cytokines might have a shared role for both autism and immunological issues through the regulation of proliferation and differentiation of neural stem cells (Bauer et al. 2007; Masi et al. 2015). In the case of autism co-occurring with GI malfunction, altered intestinal microbiota composition might impact on brain development and result in autistic behaviors either via immunological pathways or vagal nerve activation (Hsiao 2014). Conversely, via the gut-brain axis, altered brain development might also lead to dysregulated GI motility and secretion, and subsequently GI symptoms (Hsiao 2014). However, these hypotheses remain largely unsupported, and for many models the causal influence of somatic health issues on autism are unclear.

To determine the role of co-occurring somatic health problems for the emergence of autism phenotypes across the full range of behavior expression from single traits to clinical expressions of ASD, studies investigating monozygotic twins discordant for autism and autistic traits are powerful to elucidate the role of environmental risk factors, such as co-occurring somatic conditions (Losh et al. 2012). The primary reason is that ASD exhibit vast heterogeneity both at the behavioral and clinical phenotype as well as the genetic level. Hundreds of genes affected by rare large effect size variants as well as combination of common variants contribute to etiology of this disorder. Additionally, with-in family heterogeneity, variable penetrance and expressivity contribute to the complexity of genetic mechanisms in ASD (Vorstman et al. 2017). Compared to other designs, the twin design using monozygotic twins implicitly controls for genetics and other common important confounders, such as age, gender, shared environment, and family background. Thus the twin design will illuminate features of variation that are not confounded by the sample heterogeneity related to these issues. So far, to the best of our knowledge, no other study has addressed somatic health in autism using this informative approach. Therefore, this study aimed to use a twin cohort enriched for ASD and other neurodevelopmental disorders (NDDs) to explore the presence of somatic health conditions in twin pairs who are qualitatively (for ASD diagnosis) discordant or quantitatively (for autistic traits) differing for autism phenotypes (Constantino and Todd 2003). Additionally, we investigated the association between somatic health, on one hand, and ASD diagnosis as well as autistic traits, on the other, using a co-twin control design to address genetic influence and other possible shared confounds on their relation. Since individuals with ASD frequently have comorbid attention-deficit/hyperactivity disorder (ADHD) (Abdallah et al. 2011; Oerlemans et al. 2016; Simonoff et al. 2008), which may be also associated with somatic problems (Akmatov et al. 2019; Park et al. 2017), we included ADHD as a covariate in our analyses.

## Methods

### Participants

**Fig. 1:**
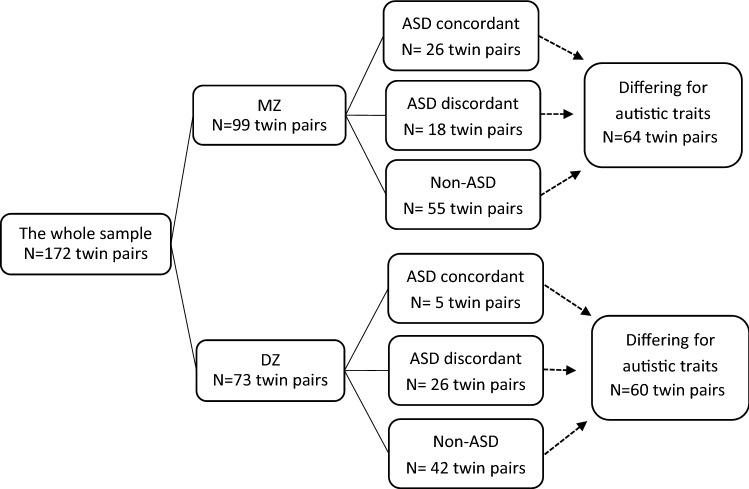
Twin pairs in analyses of the association between somatic comorbidity and clinical (ASD) and quantitative (autistic traits) autism phenotypes

Sample composition and characteristics are summarized in Fig. 1 and Table 1. The sample is part of the Roots of Autism and ADHD Twin Study Sweden (RATSS) (Bölte et al. 2014) with recruitment from August 2011 to September 2018. The sources of twins in RATSS include (a) the Child and Adolescent Twin Study in Sweden (CATSS), a nationwide population-based twin study conducted in Sweden since 1994 (Anckarsäter et al. 2011) (45.5%); (b) Swedish national registry data from the Swedish Board of Health and Welfare, clinical recruitments from services in Region Stockholm (the Division of Child and Adolescent Psychiatry, the Habilitation and Health centers, and pediatric units); (c) summons in the journals, media, autism societies, and twin organizations. Participants in RATSS are twins discordant or concordant for ASD and other NDDs, as well as typically developing (TD) twin controls. In the present study, a total of 344 twins from 172 pairs were studied, including 99 monozygotic (MZ) pairs and 73 dizygotic (DZ) pairs (mean age = 16.56 ± 5.62 years, range 8 to31). Zygosity was determined by DNA testing with saliva or whole-blood sample using standard methods as described earlier (Stamouli et al. 2018; Willfors et al. 2017). There were 44 twin pairs discordant for a primary clinical ASD diagnosis (18 MZ pairs and 26 DZ pairs), and 31 twins were concordant for a primary clinical ASD diagnosis (26 MZ pairs and 5 DZ pairs). Additionally, 124 twins were significantly differing for autistic traits (64 MZ pairs and 60 DZ pairs), as defined by an intra-pair difference on the total score of the Social Responsiveness Scale-2 (SRS-2) of at least 6 points, corresponding to 1 standard error of measurement, while a difference in total scores of 10 was considered by the authors as also being of clinical significance (Constantino and Gruber 2012). Seventy-seven percent of the pairs with differing autistic traits had a total score difference of 10 or more. The distributions for quantitative autistic trait scores in the various groups are displayed in Table 1. For the MZ pairs discordant for ASD diagnosis, the mean SRS-2 total score of the affected twins were 83.61 ± 30.37, while the score of co-twins were 33.50 ± 22.04. For the MZ pairs differing for autistic traits, the twins with higher level of autistic trait had the mean SRS-2 total score as 60.61 ± 35.44, and their co-twins had score as 33.39 ± 28.19. The current study was approved by the Regional Swedish Ethical Review Board in Stockholm and informed consents were obtained from all participants and/or their legal guardians.

**Table 1:** Sample characteristics

	Total sample		Sample for within-pair difference analyses								
			MZ discordant for ASD diagnosis		Matched typically developed MZ	DZ discordant for ASD diagnosis		Twins significantly differing for autistic traits			
								MZ		DZ	
N pairs	172		18		18	26		64		60	
Age											
Mean	16.56		15.83		16.22	14.96		16.70		15.63	
SD	5.62		4.93		4.67	5.00		5.51		5.36	
Range	8–31		9–28		10–28	8–31		8–29		8–31	

	ASD	Non-ASD	ASD twin	Co-twin		ASD twin	Co-twin	Higher SRS-2 score	Lower SRS-2 score	Higher SRS-2 score	Lower SRS-2 score
N subjects	81	263	18	18	36	26	26	64	64	60	60
Sex, N subjects											
Male	50	131	20		20	17	15	62		34	29
Female	31	132	16		16	9	11	66		26	31
NDD diagnosis, N subjects											
ASD	81	0	18	0	0	26	0	29	12	26	4
ADHD	39	56	7	2	0	12	6	19	15	31	16
ID	15	5	3	1	0	5	0	9	2	6	0
Other NDDs	23	32	5	1	0	9	3	13	7	16	8
Full-scale IQ											
Mean	91.91	100.43	91.41	98.50	102.63	93.00	100.68	93.24	97.72	96.05	102.44
SD	19.17	14.43	24.41	15.58	4.67	17.51	12.64	18.61	16.34	18.15	11.45
Range	40–142	62–138	40–138	65–121	86–123	65–127	77–128	40–138	62–142	68–138	74–128
SRS-2 total score											
Mean	86.05	29.88	83.61	33.50	19.14	84.00	31.08	60.61	33.39	72.40	30.40
SD	27.92	25.01	30.37	22.04	12.83	25.11	22.61	35.44	28.19	34.50	26.85
Range	21–141	0–142	21–131	4–93	0–65	39–124	2–87	9–131	2–117	10–142	0–120
ADI-R total A score											
Mean	12.79	2.06	12.06	5.17	1.23	11.46	1.73	7.60	4.94	7.13	1.95
SD	7.29	2.81	7.53	4.71	1.73	8.19	2.74	6.96	5.57	7.79	3.70
Range	0–30	0–15	0–26	0–14	0–6	0–28	0–11	0–26	0–21	0–28	0–20
ADI-R total B score											
Mean	9.35	1.55	8.76	4.39	0.80	7.50	1.23	5.45	3.79	5.41	1.78
SD	5.29	2.13	4.97	3.18	1.89	5.83	2.03	5.45	4.38	5.78	3.18
Range	0–22	0–10	2–18	0–10	0–8	0–21	0–8	0–19	0–19	0–22	0–15
ADI-R total C score											
Mean	3.45	0.48	3.47	0.78	0.34	3.12	0.50	1.98	0.92	2.07	0.60
SD	2.12	1.02	2.43	1.35	0.76	2.10	1.27	2.27	1.43	2.10	1.59
Range	0–10	0–5	0–8	0–5	0–3	0–9	0–5	0–8	0–5	0–9	0–10
ADOS-2 comparison scores											
Mean	6.27	1.68	5.28	2.00	1.47	6.35	2.04	3.60	2.57	3.80	1.81
SD	2.35	1.14	2.27	1.46	0.84	2.38	1.59	2.74	2.35	2.68	1.73
Range	1–10	1–7	2–9	1–7	1–4	2–10	1–7	1–10	1–9	1–10	1–9

*ASD* autism spectrum disorder, *ADHD* attention-deficit/hyperactivity disorder, *ID* intellectual disability, *NDD* neurodevelopmental disorder, *ADI*-*R* the Autism Diagnostic Interview-Revised, *ADOS* the Autism Diagnostic Observation Schedule, *SRS*-*2* social responsiveness scale, Second Edition, *MZ* monozygotic twins, *DZ* dizygotic twins

### Diagnostic and behavioral assessments

Participants received a comprehensive psychodiagnostic assessment administered by a team of experienced clinicians (see Bölte et al. 2014, for details). Briefly, diagnoses were based on the Diagnostic and Statistical Manual of Mental Disorders, Fifth Edition (DSM-5) (American Psychiatric Association 2013), supported by results from a battery of well-validated instruments, including the Autism Diagnostic Interview-Revised (ADI-R) (Rutter et al. 2003), the Autism Diagnostic Observation Schedule Second Edition (ADOS-2) (Lord et al. 2012), and the Kiddie Schedule for Affective Disorders and Schizophrenia- Present and Lifetime Version (K-SADS-PL) (Kaufman et al. 1997). Full-scale IQ was measured by using the Wechsler Intelligence Scales for Children or Adults, Fourth Editions (Wechsler 2003, 2008). Autistic traits were evaluated with the parent-report version of the SRS-2, consisting of 65 items, assessing autistic traits in terms of social communication, awareness, motivation, cognition and behaviour flexibility within the past 6 months. Items were rated on a 4-point Likert scale (ranging from 0 to 3), with higher scores indicating more autistic traits. SRS-2 raw scores were used, as recommended for research settings.

### Medical history and present somatic comorbidity

The information on medical history and present somatic health issues was collected from a questionnaire completed by the participants or their parents. The questionnaire consisted of one open question “Has the child (Have you) been seriously ill during his/her (your) childhood?” and 33 closed questions asking if the participants had ever had a specific somatic health diagnosis or problem. The full list of somatic conditions that we obtained from the questionnaire and the distribution of these conditions in our sample are summarized in Table 2. The results of the questionnaire were cross-validated with information from the “sicklist” in the Child and Adolescent Twin Study in Sweden (CATSS) (Anckarsäter et al. 2011), a population-based cohort containing more than 32,000 twins, which 250 twins also participated in the current study. The sicklist was administered as part of a neurodevelopmental symptoms assessment by telephone interview that has successfully underwent several validations (Hansson et al. 2005; Larson et al. 2010, 2013; Marland et al. 2017). Ten items of the sicklist inquiring about medical conditions overlap with our somatic health questionnaire were selected to examine the validity of our data. The agreements between these two information sources (range 75.2–99.6%) were excellent in concerning serious medical diseases, such as epilepsy (agreement = 99.6%, Kappa = 0.89, p < 0.001), and satisfactory for common symptoms like headache (agreement = 75.2%, Kappa = 0.34, p < 0.001). The comorbid somatic health issues were categorized into several physical systems (immune dysregulation, gastrointestinal problems, neurological problems, infectious disease, and cardiovascular diseases). Different conditions in the same physical system were added up to generate a predictive estimate for ASD diagnosis or autistic traits. Among these conditions, we combined the items “migraine” and “headache” into one condition “headache” and coded as one somatic health issue since the results of these two items were highly correlated.

**Table 2:** Comparisons of physical problems between ASD and non-ASD

	Total sample					MZ discordant for ASD diagnosis and TD controls					
	ASD	Non-ASD	*T*	*P* *(unadjusted)*	Cohen’s *d*	ASD twin	Co-twin (Non-ASD)	Typically developed controls (TD)	*F*	*P* *(unadjusted)*	Bonferroni post hoc tests
Somatic systems	N = 81	N = 263				N = 18	N = 18	N = 36			
Infectious diseases											
*Sum of diseases for each individual, mean*	*1.70*	*1.49*	*1.64*	*0.104*	*0.22*	*1.56*	*1.72*	*1.53*	*0.23*	*0.796*	*ASD, N*-*ASD*^*ns*^; *ASD, TD*^*ns*^*; N*-*ASD, TD*^*ns*^
Measles, n (%)	0	5 (1.9)				0	0	0			
Mumps, n (%)	0	4 (1.5)				0	0	0			
Encephalitis, n (%)	1 (1.2)	2 (0.8)				1 (5.6)	0	0			
Chickenpox, n (%)	73 (90.1)	238 (90.5)				12 (66.7)	15 (83.3)	36 (100)			
Pertussis, n (%)	7 (8.6)	20 (7.6)				1 (5.6)	3 (16.7)	2 (5.6)			
Lyme disease, n (%)	2 (2.5)	12 (4.6)				0	0	2 (5.6)			
Scarlet fever, n (%)	10 (12.3)	12 (4.6)				3 (16.7)	3 (16.7)	3 (8.3)			
Frequent cold, n (%)	20 (24.7)	32 (12.2)				5 (27.8)	4 (22.2)	4 (11.1)			
Frequent ear infection, n (%)	21 (25.9)	56 (21.3)				5 (27.8)	5 (27.8)	7 (19.4)			
RS virus infection, n (%)	2 (2.5)	6 (2.3)				0	0	0			
Pneumonia, n (%)	2 (2.5)	5 (1.9)				1 (5.6)	1 (5.6)	0			
Neurological problems											
*Sum of diseases for each individual, mean*	*0.70*	*0.38*	***2.86***	0.005*	***0.40***	*1.22*	*0.28*	*0.47*	***6.81***	0.002*	*ASD *> *N*-ASD*; ASD > TD*; N-*ASD, TD*^***ns***^
Epilepsy, n (%)	7 (8.6)	1 (0.4)				3 (16.7)	0	0			
Vertigo, n (%)	6 (7.4)	9 (3.4)				4 (22.2)	0	0			
Headache, n (%)	26 (32.1)	49 (18.6)				8 (44.4)	2 (11.1)	12 (33.3)			
Brain injury, n (%)	10 (12.3)	28 (10.6)				5 (27.8)	2 (11.1)	4 (11.1)			
Hearing impairment, n (%)	8 (9.9)	12 (4.6)				2 (11.1)	1 (5.6)	1 (2.8)			
Gastrointestinal problems											
*Sum of diseases for each individual, mean*	*0.25*	*0.17*	*1.09*	*0.278*	*0.16*	*0.28*	*0.33*	*0.22*	*0.27*	*0.767*	*ASD, N*-*ASD*^*ns*^; *ASD, TD*^*ns*^*; N*-*ASD, TD*^*ns*^
Lactose intolerance, n (%)	10 (12.3)	28 (10.6)				2 (11.1)	3 (16.7)	5 (13.9)			
Gluten intolerance, n (%)	2 (2.5)	2 (0.8)				0	0	0			
Irritable bowel, n (%)	2 (2.5)	4 (1.5)				1 (5.6)	0	0			
Diarrhea, n (%)	0	2 (0.8)				0	0	0			
Constipation, n (%)	1 (1.2)	2 (0.8)				0	0	0			
Stomach problems, n (%)	2 (2.5)	3 (1.1)				1 (5.6)	0	1 (2.8)			
Other GI problems, n (%)	3 (3.7)	4 (1.5)				1 (5.6)	2 (11.1)	0			
Immunological problems											
*Sum of diseases for each individual, mean*	*0.94*	*0.62*	***2.89***	0.004*	*0.35*	*0.94*	*1.28*	*0.72*	*1.65*	*0.199*	*ASD, N*-*ASD*^*ns*^; *ASD, TD*^*ns*^*; N*-*ASD, TD*^*ns*^
Asthma, n (%)	18 (22.2)	46 (17.5)				6 (33.3)	6 (33.3)	2 (5.6)			
Eczema, n (%)	28 (34.6)	59 (22.4)				6 (33.3)	6 (33.3)	8 (22.2)			
Milk allergy, n (%)	4 (4.9)	11 (4.2)				0	1 (5.6)	2 (5.6)			
Allergy to specific allergen, n (%)	22 (27.2)	46 (17.5)				3 (16.7)	7 (38.9)	9 (25.0)			
Other immunological problems, n (%)	4 (4.9)	1 (0.4)				0	0	0			
Cardiovascular diseases											
*Sum of diseases for each individual, mean*	*0.07*	*0.01*	*2.09*	*0.040*	*0.30*	*0.17*	*0.06*	*0*	*3.34*	*0.041*	*ASD, N*-*ASD*^*ns*^; *ASD, TD*^*ns*^*; N*-*ASD, TD*^*ns*^
Patent Ductus Arteriosus, n (%)	1 (1.2)	0				1 (5.6)	0	0			
Problems of aorta, n (%)	1 (1.2)	0				1 (5.6)	0	0			
Ventricular septal defect, n (%)	2 (2.5)	0				0	0	0			
Vegetations, n (%)	0	1 (0.4)				0	0	0			
Cardiomegaly, n (%)	1 (1.2)	0				0	0	0			
Unspecified heart problems, n (%)	1 (1.2)	2 (0.8)				1 (5.6)	1 (5.6)	0			

With Bonferroni correction, the significance level of p value in this table is set at 0.01. Brain injuries included hydrocephalus, cerebral palsy, intracerebral hemorrhage, congenital cerebral malformation, and concussion; other GI problems included gastroenteritis, intestinal polyps, abdominal pain, esophagitis, and biliary atresia; other immunological problems included type 1 diabetes, ankylosing spondylitis, and immunodeficiency

^ns^ non-significant, *p < 0.01

### Statistical analysis

All statistical analyses were performed with IBM SPSS software version 25 (SPSS Inc., Chicago, IL, USA) and the drgee package (Zetterqvist et al. 2016) in R version 3.2.4. Student’s t test was used to compare the prevalence of somatic comorbidity between the individuals with ASD and without ASD in the whole sample. To examine if the diagnosed twins in MZ ASD discordant pairs showed a higher prevalence of comorbid somatic conditions compared to their co-twin and TD controls, we selected age and gender matched MZ control pairs from our sample. Analysis of variance (ANOVA) was used to compare the number of somatic health problems among the diagnosed twins, their undiagnosed co-twins, and TD individuals, with post hoc testing between groups. For the co-occurring somatic health conditions which were identified with higher prevalence rates in ASD in the whole sample, conditional multivariate logistic and linear regression analysis with twin pairs clustered was used to explore the adjusted associations between somatic health and ASD diagnosis as well as autistic traits, after adjustment for potential confounding variables. The correlation between intra-pair differences in SRS-2 total score and somatic health problems in twin pairs significantly differing for autistic traits was tested by their Pearson correlations (r). To determine the within pair effect of somatic comorbidity on the development of autism among discordant/quantitatively differing twins, we used a statistical framework of multiply adjusted (conditional) linear regressions based on generalized estimation equations (GEE) in order to fully account for its specific premises, and allowing both categorical/clinical and dimensional/trait autism outcomes. All tests were two-tailed and p-values of 0.05 or less were considered statistically significant. A Bonferroni correction was made for multiple comparisons in all the analyses.

## Results

### Comparisons of physical problems between ASD and non-ASD

In the whole twin sample, participants with ASD diagnosis had significantly more neurological (t = 2.86, p = 0.005) and immunological health problems (t = 2.89, p = 0.004) compared to those without ASD (Table 2). There was no difference found for infectious diseases and gastrointestinal problems between participants diagnosed with ASD and non-ASD twins. Comparisons within pairs between MZ twins discordant for ASD diagnosis and matched TD controls revealed that twins with ASD had significant more neurological health problems than their co-twins and controls (F = 6.81, p = 0.002), while there was no difference between typically developing co-twins and controls. There was no difference among these three groups regarding infectious diseases, immunological problems, and gastrointestinal problems. On the other hand, there was a trend that participants with ASD had more cardiovascular health issues than those without ASD, both in the whole sample and the MZ twins discordant for ASD (p = 0.040 and p = 0.041, respectively).

### The association between somatic comorbidity and autism severity for the whole sample

Neurological conditions were significantly associated with the severity of autistic traits as measured with SRS-2, even when controlling for the possible confounders (β = 5.60, p = 0.020), such as a comorbid diagnosis of ADHD, other NDDs, and level of IQ (Table 3a). However, there was no significant association between neurological conditions and ASD diagnosis (β = 0.40, p = 0.061). For immunological conditions, we found significant association with ASD diagnosis (β = 0.43, p = 0.014), but not with autistic traits (β = 3.76, p = 0.048). We separately, examined the confounding effects of fetal distress and umbilical cord complications, which could increase the risk of hypoxic encephalopathy, in our analysis for the whole sample (see Table S1 in the Supplemental Material). The association between neurological problems and autistic traits became non-significant, while the immunological problems remained significantly associated with ASD diagnosis. However, since there could also be high collinearity between perinatal hypoxic insults and the subsequent neurological complications, we did not include perinatal insults as a covariate in our analysis. The analysis of the association between neurological problems and autistic traits in ASD and in non-ASD (Table 3b) revealed a significant association in individuals with ASD diagnoses (β = 5.17, p = 0.039), but not in those without.

**Table 3:** Associations between neurological/immunological problems and ASD diagnosis and autistic traits in the whole sample

(a) The analyses in the whole sample with outcome as ASD diagnosis and autistic traits (n = 344)							
		Outcome					
		ASD diagnosis			Autistic traits		
		β	S.e.	P (unadjusted)	β	S.e.	P (unadjusted)
Exposure variable:	Neurological Problems	0.40	0.21	0.061	5.60	2.41	0.020*
Covariate 1:	Full-scale IQ	− 0.03	0.01	0.045	− 0.43	0.12	<0.001*
Covariate 2:	ADHD	0.85	0.35	0.015*	28.22	4.07	<0.001*
Covariate 3:	Gender	0.21	0.33	0.525	0.36	3.34	0.915
Covariate 4:	Age	− 0.03	0.04	0.431	− 0.84	0.29	0.004*
Covariate 5:	Other NDDs	0.79	0.40	0.048	20.56	5.59	<0.001*
Exposure variable:	Immunological Problems	0.43	0.17	0.014*	3.76	0.90	0.048
Covariate 1:	Full-scale IQ	− 0.31	0.01	0.012*	− 0.48	0.11	<0.001*
Covariate 2:	ADHD	0.92	0.35	0.008*	29.25	4.17	<0.001*
Covariate 3:	Gender	0.21	0.34	0.525	0.42	3.37	0.900
Covariate 4:	Age	− 0.02	0.03	0.491	− 0.79	0.28	0.005*
Covariate 5:	Other NDDs	0.85	0.40	0.033	21.08	5.54	<0.001*

(b) Associations between neurological problems and autistic traits in subjects with and without ASD diagnosis							
		Outcome: autistic traits					
		ASD (n = 81)			Non-ASD subjects (n = 263)		
		β	S.e.	P	β	S.e.	P
Exposure variable:	Neurological problems	5.17	2.51	0.039**	1.38	2.18	0.526
Covariate 1:	Full-scale IQ	− 0.36	0.16	0.020**	− 0.25	0.10	0.012**
Covariate 2:	ADHD	9.25	5.58	0.097	27.07	4.24	<0.001**
Covariate 3:	Gender	− 0.59	5.93	0.921	− 0.92	2.92	0.754
Covariate 4:	Age	− 0.46	0.44	0.299	− 0.81	0.21	<0.001**
Covariate 5:	Other NDDs	21.83	6.43	<0.001**	10.31	4.36	0.018**

With Bonferroni correction, the significance level of p value in this table is set at 0.025

*ASD* autism spectrum disorder, *ADHD* attention-deficit/hyperactivity disorder, *NDD* neurodevelopmental disorder

*p < 0.025; **p < 0.05

### With-in pair effect of somatic comorbidity on autism severity for diagnosis discordant/quantitatively differing twin pairs

The intra-pair differences of neurological health problems were significantly correlated with the total score differences on the SRS-2 for MZ twins differing for autism traits (r = 0.40, p = 0.001, Table 4). In the conditional logistic model, twins with more neurological problems were more likely to have an ASD diagnosis than their co-twins in the MZ ASD-discordant twin pairs (Odds ratio per problem [Confidence interval 95%] = 3.15 [1.20–8.30], p = 0.020, Table 5a). In addition, for the MZ quantitatively differing twins, within-twin pair increases in neurological problems were associated with increases in SRS-2 total scores (β = 10.44, p = 0.006, Table 5b).

**Table 4:** The correlation between intra-pair differences in autistic traits and somatic problems in MZ and DZ twin pairs significantly differing for autistic traits

Difference in somatic problems	Differing for autistic traits			
	MZ, n = 64 pairs		DZ, n = 60 pairs	
	R	P (unadjusted)	R	P (unadjusted)
Infectious disease	0.047	0.711	− 0.040	0.759
**Neurological problems**	**0.399**	0.001*	0.017	0.898
GI problems	− 0.114	0.369	0.084	0.522
Immunological problems	− 0.113	0.372	0.176	0.178
Cardiovascular diseases	0.111	0.384	–	–

With Bonferroni correction, the significance level of p value in this table is set at 0.01

*p < 0.01

**Table 5:** Associations between neurological problems and ASD diagnosis/autistic traits and in MZ and DZ twin pairs discordant for ASD and significantly differing for autistic traits

(a) Discordant for ASD diagnosis							
		Outcome: ASD diagnosis					
		MZ, n = 18 pairs			DZ, n = 26 pairs		
		β	S.e.	P	β	S.e.	P
Exposure variable:	Neurological problems	1.15	0.49	0.020*	− 0.07	0.40	0.854
OR per neurological problem (95% CI)		**3.15 (1.20**–**8.30)**			0.93 (0.42–2.04)		

(b) Differing for autistic traits							
		Outcome: autistic traits					
		MZ, n = 64 pairs			DZ, n = 60 pairs		
		β	S.e.	P	β	S.e.	P
Exposure variable:	Neurological Problems	10.44	3.76	0.006*	4.79	7.28	0.510
Covariate 1:	Full-scale IQ	−1.10	0.30	< 0.001*	− 0.73	0.31	0.018*
Covariate 2:	ADHD	12.23	9.40	0.193	34.69	7.73	< 0.001*
Covariate 3:	Other NDDs	14.85	8.63	0.085	28.68	9.70	0.003*
Covariate 4:	Gender	–	–	–	24.83	8.73	0.004*

*ASD* autism spectrum disorder, *ADHD* attention-deficit/hyperactivity disorder, *NDD* neurodevelopmental disorder

*p < 0.05

## Discussion

This is the first study to investigate the association of autism and somatic health and the impact of somatic health issues on clinical autism phenotypes as well as autistic traits in a large twin sample enriched for ASD and other NDDs. With the co-twin control design, our results demonstrated a significant within pair effect of neurological health issues on both clinical ASD diagnosis and autistic traits, but no similar effects were observed on autism by any other health problem in other physical systems. Our findings suggest that neurological health problems can be considered non-shared environmental factors associated with autistic traits and clinically relevant expressions of autism acting in concert with other contributing factors such as genetic background. These results also endorse the conceptualization of ASD as a neurodevelopmental condition which often involves some form of altered brain development (Piven et al. 2017). To further enhance our understanding of possible biological autism subtypes and their impact, future research should explore the impact of neurological conditions in these strata on symptom profiles and social functioning and impairment in a given context.

The prevalence of other conditions previously associated with ASD and common physical problems were examined in our study population and yield inconsistent results. While being in line with previous prevalence studies regarding higher rates of comorbid neurological problems and immunological dysregulations (Davignon et al. 2018; Kohane et al. 2012; Schendel et al. 2016; Schieve et al. 2012), our data revealed no evidence for an increase of infectious diseases and congenital cardiovascular abnormalities in autism (Alexeeff et al. 2017; Timonen-Soivio et al. 2015). However, the trend that individuals with autism were prone to have congenital heart malformations (non-significant after the Bonferroni correction) might be associated with somatic pleiotropy of ASD-related genetic variants (Vorstman et al. 2017). We did not find higher rates of GI problems in participants with ASD, in contrast to the results of a prior meta-analysis (McElhanon et al. 2014). There are several aspects which need to be considered for this discrepancy. First, although GI symptoms are frequently noted among children with ASD, most of them are idiopathic (Kang et al. 2014; Xinias and Mavroudi 2015). In addition, constipation and abdominal pain in children with ASD might be associated with toileting problems, changes in toileting routine, severe food selectivity, and also emotional symptoms thus being transient rather than long-term problems (Borowitz et al. 2003; Philips et al. 2015; Volkert and Vaz 2010). Therefore, point prevalence and period prevalence of these comorbid GI problems may vary substantially across studies with different methodology (Holingue et al. 2018). Second, the prevalence of GI comorbidity among ASD patients may change along the lifespan (Vohra et al. 2017; Wise et al. 2017). As our design is well controlled for major confounders, including effects of age and genetics, it might be concluded that results of previous literature might be accounted for by other factors than primarily autism specific ones.

Conditional regression analyses showed that neurological problems were associated with the severity of autistic traits in our whole sample. This is in line with the results of prior research, where individuals with neurological conditions displayed more challenges with social communication and facial emotion recognition compared to those without, independent of intellectual disability (Ko et al. 2016; Richard et al. 2017). Our results support the hypothesis that common neurological alterations impact on social brain networks to generate autistic phenotypes (Richard et al. 2017). Furthermore, the neurological conditions in our analysis consisted of wide range of diagnoses and symptoms, including relatively minor symptoms like headache, which has also been found in ASD in previous studies (Gurney et al. 2006; Schieve et al. 2012). This is consistent with the notion of autism not only being a neurodevelopmental concept along a continuum of behavioural traits, but perhaps also neurological issues from mild to severe in the clinical spectrum of autistic traits. Our findings also indicate that a global neurological assessment for individuals with ASD is needed since both minor neurological symptoms and specific neurological issues might be associated with autistic traits and thus require adequate evaluation, study, and management. On the other hand, the association between neurological problems and autistic traits was not significant in non-ASD subjects. This might suggest that neurological complications are more likely to compound autistic traits in particular circumstances, which could be a certain level of alterations of the neural substrates associated with ASD. It also supports the notion that the underpinning of autistic phenotypic variation forms a complex interplay of synergic effects and multiple factors, including those of genetic susceptibility and environmental adversities.

The results of conditional regression analyses also revealed that immunological health issues are associated with ASD diagnosis, but not autistic traits. Consistent with previous epidemiological studies with representative samples, ASD are associated with asthma, eczema, and a wide range of allergic conditions (Chen et al. 2013; Xu et al. 2018). However, although several investigations have reported that altered serum cytokine levels may be associated with ASD symptoms (Al-Ayadhi and Mostafa 2012; Ashwood et al. 2011; Hashim et al. 2013), there is still scarce information on the relationship between clinical immunological problems and autistic traits. Our findings suggest that immunological dysregulation might only be of significance for syndromal or some clinical variants of autism, not the whole continuum of traits. Further studies are required to clarify the mechanisms underlying the association between immunological problems and ASD, as well as impact of immunological dysregulation on the symptom presentation and therapeutic outcomes of individuals with autism.

For the monozygotic twin pairs discordant for ASD diagnosis and differing for autistic traits, neurological conditions showed significant effects on autistic trait expression. With the co-twin control design, our results demonstrated that neurological alterations could be a non-shared environmental factor that contributes to autistic features independently of the genetic influence and shared prenatal and perinatal adversity. Moreover, our results indicate that the etiological pathways of autism may lead to both behavioural and neurological manifestations. Overall, emerging evidence suggests that ASD is associated with alterations in brain morphology (van Rooij et al. 2018) and connectivity (Mohammad-Rezazadeh et al. 2016). However, the with-pair effects on autistic traits of neurological conditions were not found in dizygotic twins, who differ with respect to their genetic makeup. This raises the possibility that the influence of neurologic problems on the severity of autistic traits is more pronounced in those with high genetic risks or those with certain genetic backgrounds than in other contexts. Moreover, as in our study, the autism findings are far from being universal and detected differences limited when using a whole group approach. Hence, as pointed out previously, to identify more homogenous subgroups of ASD with more clear somatic health profiles might be a pathway to better healthcare for this population compared to an unspecific universal approach to autism intervention.

There are several potential limitations to this study. First, our sample may not be representative in terms of the prevalence of somatic comorbidity in ASD due to the sampling and different source of recruitment. In addition, the sample size of twins discordant for ASD diagnosis was relatively small. Second, information on somatic comorbidity was obtained through self/parent-administered questionnaires, which are susceptible to reporting and recall bias. Our finding might be limited since the agreement between different information sources was modest for some somatic problems. Since medical records may not cover minor physical symptoms, validated instruments for identifying somatic problems or clinical interviews by physicians should be considered in future studies to improve the validity of data. Third, the results of this study might also be limited due to an unweighted approach. We were unable to estimate the possible association of each somatic with autistic features. Fourth, the causal relationship between neurological comorbidity and ASD could not be clarified with our study design. For example, some of the neurological symptoms, such as headache, might be secondary to medication or other origins. Fifth, as autism is defined as a neurodevelopmental condition, and only a few individuals on the autism spectrum or other NDDs regularly receive full physical check-ups, avoid health care services or experience insufficient treatment for somatic health concerns, somatic health issues might be overshadowed, not be detected or correctly diagnosed in this group.

## Conclusions

Our findings suggest that increased amounts of neurological problems are quite typical of autism, and also associated with the intensity of autistic traits. They furthermore indicate that neurological health issues form a non-shared environmental factor for autism phenotypes. Thus, overall, our results endorse the conceptualization of ASD in terms of a neurodevelopmental condition. To provide better healthcare for individuals with ASD and somatic health issues, it is crucial to identify these subgroups with specific biological profiles, and to develop targeted treatments addressing their symptoms and etiology. In addition, we found that ASD diagnosis was associated with immunological problems compared to those without ASD, in line with previous prevalence studies. However, there was no significant association between immunological dysregulation and the severity of autistic traits in our study.

## Electronic supplementary material

Below is the link to the electronic supplementary material.

Supplementary material 1 (DOCX 75 kb)
