# Supplementary material for: The Association Between Somatic Health, Autism Spectrum Disorder, and Autistic Traits
Source: Behav Genet. 2019 Dec 6;50(4):233–46. doi: 10.1007/s10519-019-09986-3 (PMC7355269; doi:10.1007/s10519-019-09986-3)
Supplement: Supplementary file 1 — Supplementary material 1 (DOCX 75 kb) [file 10519_2019_9986_MOESM1_ESM.docx]

**Supplemental materials**

Figure S1. Intra-pair differences in autistic traits in monozygotic twins


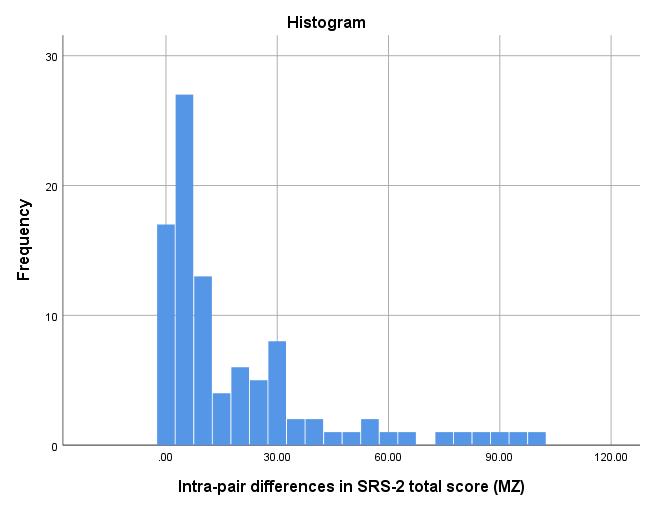


Figure S2. Intra-pair differences in autistic traits in dizygotic twins


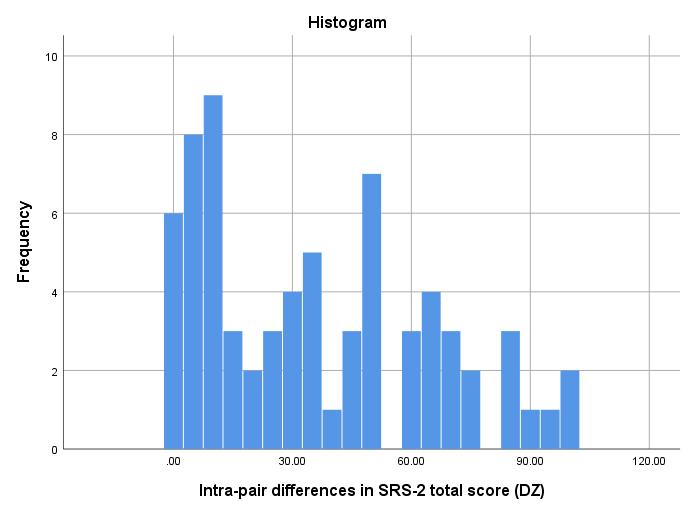


Table S1. The analyses in the whole sample with outcome as ASD diagnosis and autistic traits (n=344), with perinatal insults as a covariate

|  | | Outcome | | | | | | |
| --- | --- | --- | --- | --- | --- | --- | --- | --- |
|  | | ASD diagnosis | | |  | Autistic traits | | |
|  |  | β | s.e. | p  (unadjusted) |  | β | s.e. | p  (unadjusted) |
| Exposure variable: | Neurological Problems | 0.36 | 0.21 | 0.089 |  | 4.98 | 2.44 | 0.041 |
| Covariate 1: | Full-scale IQ | −0.03 | 0.01 | 0.045 |  | −0.43 | 0.11 | **<0.001*** |
| Covariate 2: | ADHD | 0.86 | 0.35 | **0.013*** |  | 28.41 | 4.05 | **<0.001*** |
| Covariate 3: | Gender | 0.19 | 0.34 | 0.572 |  | −0.11 | 3.28 | 0.972 |
| Covariate 4: | Age | −0.03 | 0.04 | 0.463 |  | −0.82 | 0.29 | **0.004*** |
| Covariate 5: | Other NDDs | 0.81 | 0.40 | 0.042 |  | 20.76 | 5.62 | **<0.001*** |
| Covariate 6: | Perinatal insults | 0.36 | 0.40 | 0.363 |  | 6.33 | 4.39 | 0.149 |
|  |  |  |  |  |  |  |  |  |
| Exposure variable: | Immunological Problems | 0.41 | 0.17 | **0.016*** |  | 3.38 | 1.86 | 0.069 |
| Covariate 1: | Full-scale IQ | −0.03 | 0.01 | **0.015*** |  | −0.47 | 0.11 | **<0.001*** |
| Covariate 2: | ADHD | 0.94 | 0.35 | **0.007*** |  | 29.33 | 4.14 | **<0.001*** |
| Covariate 3: | Gender | 0.19 | 0.34 | 0.586 |  | −0.10 | 3.32 | 0.975 |
| Covariate 4: | Age | −0.02 | 0.03 | 0.527 |  | −0.77 | 0.28 | **0.006*** |
| Covariate 5: | Other NDDs | 0.87 | 0.40 | 0.031 |  | 21.24 | 5.56 | **<0.001*** |
| Covariate 6: | Perinatal insults | 0.36 | 0.37 | 0.334 |  | 7.05 | 4.19 | 0.092 |

With Bonferroni correction, the significance level of p value in this table is set at 0.025.

* p < 0.025
